# Mining of epitopes on spike protein of SARS-CoV-2 from COVID-19 patients

**DOI:** 10.1038/s41422-020-0366-x

**Published:** 2020-07-01

**Authors:** Bao-zhong Zhang, Ye-fan Hu, Lin-lei Chen, Thomas Yau, Yi-gang Tong, Jing-chu Hu, Jian-piao Cai, Kwok-Hung Chan, Ying Dou, Jian Deng, Xiao-lei Wang, Ivan Fan-Ngai Hung, Kelvin Kai-Wang To, Kwok Yung Yuen, Jian-Dong Huang

**Affiliations:** 1 CAS Key Laboratory of Quantitative Engineering Biology, Shenzhen Institute of Synthetic Biology, Shenzhen Institutes of Advanced Technology, Chinese Academy of Sciences, Shenzhen, Guangdong 518055 China; 2 School of Biomedical Sciences, Li Ka Shing Faculty of Medicine, University of Hong Kong, 3/F, Laboratory Block, 21 Sassoon Road, Hong Kong, China; 3 Department of Medicine, University of Hong Kong, 4/F Professional Block, Queen Mary Hospital, 102 Pokfulam Road, Hong Kong, China; 4 Department of Microbiology, University of Hong Kong, 19/F T Block, Queen Mary Hospital, 102 Pokfulam Road, Hong Kong, China; 5 Beijing Advanced Innovation Centre for Soft Matter Science and Engineering (BAIC-SM), College of Life Science and Technology, Beijing University of Chemical Technology, Beijing, 100029 China

**Keywords:** Immunology, Mechanisms of disease

Dear Editor,

The ongoing coronavirus disease 2019 (COVID-19) pandemic caused by severe acute respiratory syndrome coronavirus-2 (SARS-CoV-2) is a serious threat to global public health, and is imposing severe burdens on human society. Several candidate vaccines against SARS-CoV-2 are now undergoing clinical trials. The Spike (S) protein of SARS-CoV-2 is widely considered as a promising antigen. However, limited information about the protective immune response against SARS-CoV-2 has been reported.^1^ In vivo or in natura data of the immune response in patients, including major immune responses to S protein, are currently lacking. The development of effective and safe vaccines against SARS-CoV-2 is urgently needed because of some potential adverse events including antibody-dependent enhancement (ADE),^2^ which might be difficult to avoid in current vaccine designs. Therefore, it is important to mine serological information from COVID-19 patients. In this study, we analysed the correlation between S- or Nucleocapsid (N) protein-specific antibody levels and neutralizing antibody tires. Furthermore, we aimed to identify linear B cell linear immunodominant (ID) sites on the S protein by Pepscan analysis with a series of overlapped peptides against the sera from COVID-19 patients.

We profiled IgG/IgM/IgA levels against the S and N proteins in the sera of COVID-19 patients (Supplementary information, Fig. S1a–f). All serum samples from COVID-19 patients tested positive for SARS-CoV-2 were assayed by ELISA using plates coated with SARS-CoV-2 lysates (Fig. 1a). All convalescent sera from the COVID-19 patients contained specific IgG antibodies against recombinant SARS-CoV-2 N protein, but not all hospitalized patient sera had specific IgG antibodies for the RBD fragment of the S protein due to their early infection stage. The relatively high immunogenicity of SARS-CoV-2 N protein during infection showed it has potential as an antigen for developing COVID-19 diagnostics (Supplementary information, Fig. S1d–f). However, the amounts of the different antibodies varied across patients. We found that IgM contributed 5%–34% of N protein-specific antibodies, whereas anti-RBD IgM contributed 10%–49% of RBD-specific antibodies (Supplementary information, Fig. S1g, h).

**Fig. 1:**
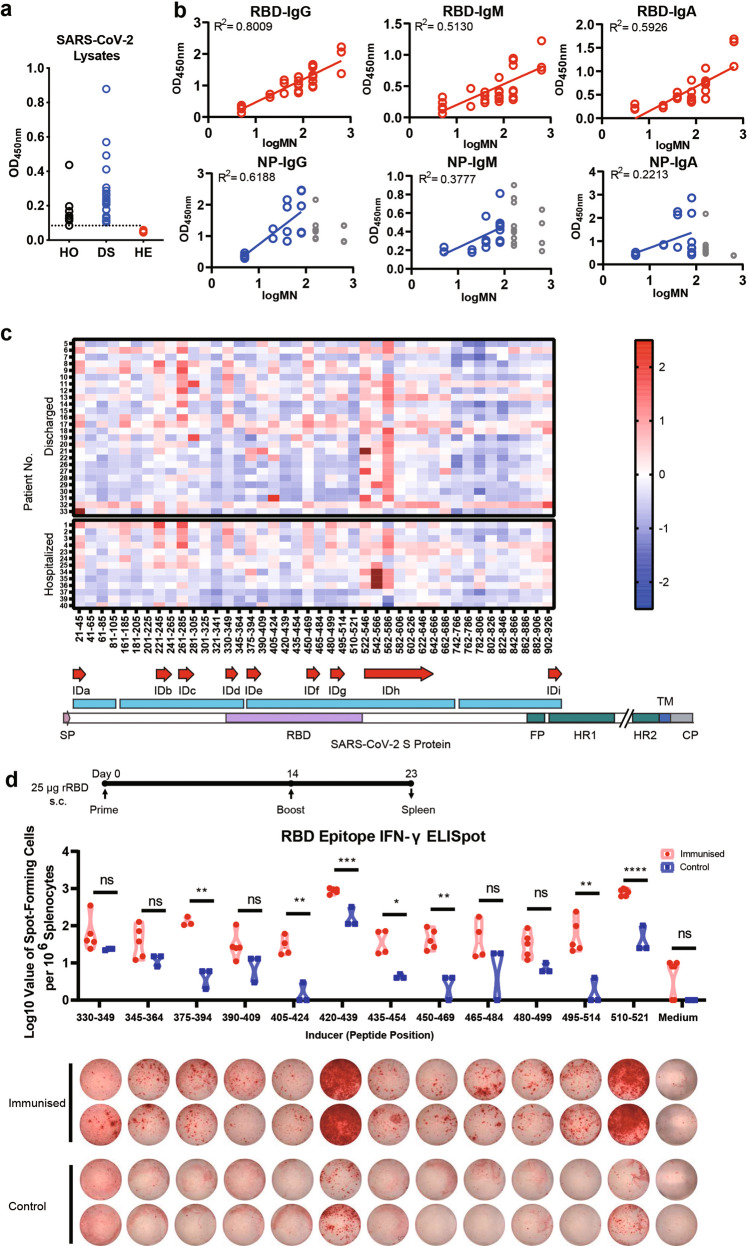
Detecting immune responses in COVID-19 patients and mining epitopes on spike protein of SARS-CoV-2. **a** Total proteins from SARS-CoV-2 lysates were used as the coated antigen. Sera from 26 discharged patients, 13 hospitalized patients, and 6 healthy blood donors were tested at a dilution of 1:100. The dashed lines represent cut-off values (the mean absorbance at 450 nm of sera from healthy blood donors plus three times the standard deviation). HO: Hospitalized patients’ sera, DS: Discharged patients’ sera, HE: Healthy donors’ Sera. **b** Correlation between N protein or RBD fragment of S protein-specific IgM levels and microneutralisation antibody titres. To compare different correlations, the MN titres were adjusted following previous criteria: MN titres less than 10 were re-designated a value of 5 and MN titres greater than 320 were re-designated a value of 640. **c** The landscape of adjusted epitope-specific antibody levels in each patient and schematic representation of SARS-CoV-2 S protein and identified B cell immunodominant sites. The ELISA results of absorbance at 450 nm were normalized to the aforementioned cut-off values. Here, only epitopes with positive rates greater than 50% are immunodominant. **d** IFNγ-ELISpot result for T cell immunodominant sites in mouse. Balb/C mice (*n* = 5 per group) were immunised subcutaneously (s.c.) with 25 µg of rRBD mixed with aluminum hydroxide gel (AHG). Number of IFNγ-secreting splenocytes in response to stimulation with the 12 RBD peptide pools of 20-mer peptides. Student’s *t*-test was used with multiple *t*-tests adjustment. Data were expressed as mean ± SD. *P < 0.05, **P < 0.01, ***P < 0.001, ****P < 0.0001, ns not significant.

We also analysed the correlation between S or N protein-specific antibody levels and SARS-CoV-2 neutralizing titres by adjusting the microneutralisation (MN) titres (Supplementary information, Fig. S1i–k). We identified a very strong correlation between anti-RBD IgG titres and MN activity in recovered patients (R^2^ = 0.8009). The correlations between anti-RBD IgM/IgA titres and MN activity were weaker than for IgG (R^2^ = 0.5130 and 0.5926, respectively) (Fig. 1b). This observation indicates that RBD-specific antibodies in the sera of recovered patients might provide antiviral protection mainly through neutralizing rather than non-neutralizing antibody activity against the N protein. This suggests that manipulating the RBD-induced immune responses might have the potential to be used in developing more effective COVID-19 vaccines.

Earlier research identified five linear ID sites in the S protein of SARS-CoV in 2005.^3^ However, in 2010s, research began to make the connection between ID sites and potential risk factor, ADE.^4^ Thus, it is crucial to map the immunogenicity sites of potential linear or conformational epitopes of the SARS-CoV-2 S protein to accelerate the development of safe vaccines. To identify the linear B Cell ID sites on the SARS-CoV-2 S protein, we analysed the epitopes with 42 chemical synthesized peptides (Supplementary information, Table S1) spanning the entire extra-membrane domain (21–926) of the S protein with three gaps (106–160, 365–374, and 687–741). Each peptide was between 20 and 25 residues in length with a five-residue overlap. We measured ID sites in terms of the positive rate (the percentage of convalescent sera from COVID-19 patients having positive reactions to the epitopes). Here, we used the mean response plus three times the standard deviation in healthy donors as the cut-off value to define positive reactions (Supplementary information, Fig. S2). The epitope mapping revealed nine linear ID sites on the S protein located at 21–45(IDa), 221–245(IDb), 261–285(IDc), 330–349(IDd), 375–394(IDe), 450–469(IDf), 480–499(IDg), 522–646(IDh), and 902-926(IDi), respectively (Fig. 1c; Supplementary information, Fig. S3b, c), with an average positive rate of ≥ 50% among all 39 patients. We found that the SARS-CoV-2 RBD contained four ID sites, IDd, IDe, IDf, and IDg. Considering SARS-CoV-2 Spike protein shares 75.96% amino acid sequence identity with that of the SARS-CoV, we found five out of the nine fragments, IDc (79.17%), IDd (90%), IDe (90%), IDh (79.2%), and IDi (96%) of SARS-CoV-2 S protein were evolutionarily highly conserved in the SARS-CoV S protein (Supplementary information, Fig. S3a). These results suggest that the conserved regions contribute to the immunogenicity of the S protein. However, positive rates of these conserved amino acid sequences as examined by patient sera are different in SARS-CoV vs SARS-CoV-2 patients. Only three (IDa, IDh, and IDi) out of the nine SARS-CoV-2 ID sites have similar positive rates when comparied to SARS-CoV ID sites.^3^ Interestingly, while there are no ID sites in RBD fragment of SARS-CoV,^3^ four ID sites were identified in the RBD fragment of SARS-CoV-2. Several amino acid alterations (Y^442^→L^455^, L^443^→F^456^, N^479^→Q^493^) in SARS-CoV-2 might change the immunogenicity of ID sites IDf (450–469) and IDg (480–499) when compared to SARS-CoV. We also compared linear ID epitopes with conformational epitopes of previously identified SARS-CoV-2 antibodies.^5–9^ Interestingly, some binding residues, in the form of conformational epitopes, of SARS-CoV-2-specific antibodies were similar to the ID sites (Supplementary information, Fig. S3a). This suggest that linear ID epitopes may associate with conformational epitopes. However, neither conformational epitopes nor linear ID epitopes provided functional information on whether they were protective or not when used to induce immune responses in vivo. Thus, future studies are needed to identify the functions of linear or conformational epitopes.

To characterize the T-cell epitopes located in the RBD region, BALb/c mice were immunized with recombinant RBD (rRBD) protein. Overlapping 20-mer peptide pools were used to stimulate splenocytes from rRBD immune animals. Splenocytes were subsequently analysis by ELISpot for the release of IFNγ. S375–394, S405–469, and S495–521 were observed to stimulate robust secretion of IFNγ from splenocytes (Fig. 1d). The response of CD4^+^ T cell epitopes in the RBD fragment showed distinct patterns compared to that of B cells. Among the nine epitopes on the S protein, S370–394 (IDe), S450–469 (IDf), and S480–499 (IDg) were identified as linear B cell ID sites. These data suggested that S370–394, S450–469, and S480–499 epitopes are more likely to be both T and B cell linear ID sites. Synthetic peptides corresponding to B and T cell ID can induce high titres of RBD-specific antibodies in a mouse model, but these antibodies possess only weak neutralizing activity (Supplementary information, Fig. S4). We are extremely concerned about the existence of potent non-neutralizing antibodies induced by linear ID sites or other epitopes, which might have enhancing effects rather than protective effects when used as potential vaccines. Thus, the selection of epitopes in the development of a vaccine might be a trade-off between enhancing and protective effects considering both B cell and T cell responses. An ideal vaccine against SARS-CoV-2 should be de novo designed rationally based on epitopes that induce highly potent neutralizing antibodies instead of disease-enhancing antibodies.^10^

Our findings provide serological evidence of immune responses in vivo, and offer initial useful information for the use of specific antigenic epitopes of S protein instead of the entire S protein to design a vaccine against SARS-CoV-2 with better safety and higher effectiveness. Our work revealed for the first time the major linear ID sites of the SARS-CoV-2 S protein. Although continuous epitope structures might be lost, we were able to show immune responses against these linear continuous epitopes generated in natura as detected in an ex vivo assay. It will be necessary to reveal the function of all the ID epitopes including linear or conformational epitopes for the rational design of de novo peptide-based vaccines.

## Supplementary information

Supplementary Information

## Data Availability

All data used to draw the conclusions in the paper are presented in the paper and/or the supplementary materials.
