# Supplementary material for: Mining of epitopes on spike protein of SARS-CoV-2 from COVID-19 patients
Source: Cell Res. 2020 Jul 1;30(8):702–4. doi: 10.1038/s41422-020-0366-x (PMC7327194; doi:10.1038/s41422-020-0366-x)
Supplement: Supplementary file 1 — Supplementary Information [file 41422_2020_366_MOESM1_ESM.pdf]

Mining of Epitopes on Spike Protein of SARS-CoV-2 from COVID-19 Patients

Bao-zhong Zhang[1,2] , Ye-fan Hu[2,4] , Lin-lei Chen[3] , Thomas Yau[4] , Yi-gang Tong[5] , Jing-chu Hu[1], Jian-piao Cai[3], Kwok-Hung Chan[3], Ying Dou[2], Jian Deng[2], Xiao-lei Wang[2], Ivan Fan-Ngai Hung[4], Kelvin Kai-Wang To[3], Kwok Yung Yuen[3], Jian-Dong Huang[1,2]

[1] CAS Key Laboratory of Quantitative Engineering Biology, Shenzhen Institute of Synthetic Biology， Shenzhen Institutes of Advanced Technology, Chinese Academy of Sciences, Shenzhen 518055, China

[2] School of Biomedical Sciences, Li Ka Shing Faculty of Medicine, University of Hong Kong, 3/F, Laboratory Block, 21 Sassoon Road, Hong Kong, China

[3] Department of Microbiology, University of Hong Kong, 19/F T Block, Queen Mary Hospital, 102 Pokfulam Road, Hong Kong, China

[4] Department of Medicine, University of Hong Kong, 4/F Professional Block, Queen Mary Hospital, 102 Pokfulam Road, Hong Kong, China

[5] Beijing Advanced Innovation Centre for Soft Matter Science and Engineering (BAIC-SM), College of Life Science and Technology, Beijing University of Chemical Technology, Beijing 100029, China

These authors contributed equally: Bao-zhong Zhang, Ye-fan Hu, Lin-lei Chen, Thomas Yau, Yi-gang Tong

Correspondence: Kelvin Kai-Wang To (kelvinto@hku.hk) or Kwok Yung Yuen (kyyuen@hku.hk) or Jian-Dong Huang (jdhuang@hku.hk)

Supplementary Information

Materials and Methods

Serum specimens from COVID-19 patients

Serum samples were collected from 39 patients with COVID-19 (22 males and 17 females) between Jan 26, 2020, and March 18, 2020. Twenty-six patients who were discharged from hospital provided written informed consent under UW 13-265. The other 13 hospitalized patients were sampled under UW 13-372, and written informed consent was waived. Average sampling time from the onset date was 28.7 days (range, 14–44 days). The median age of patients was 59.7 years (range, 26-80 years). All patients were diagnosed with COVID-19 by PCR test, and two patients had a severe illness. The diagnostic criteria for SARS-CoV-2 infection followed the clinical description of COVID-19[1]. The initial laboratory confirmation was performed on nasopharyngeal or sputum specimens at the Public Health Laboratory Centre of Hong Kong. The discharge criteria were clinical stability and negative nucleic acid test twice consecutively (sampling interval ≥ 24 hours). Six healthy donors were also sampled under UW 19-470. This study was approved by the Institutional Review Board of the University of Hong Kong/Hospital Authority Hong Kong West Cluster (UW 13-372, UW 13-265, and UW 19-470).

Animal experiments

Specific-pathogen-free (SPF) BALB/c mice were supplied by the Laboratory Animal Unit of the University of Hong Kong. All animal experiments were approved by the Committee on the Use of Live Animals in Teaching & Research, the University of Hong Kong (CULATR 5312-20). The recombinant S protein RBD or peptide was formulated at a ratio of 9:1 in aluminium hydroxide gel (Alhydrogel, AHG, InvivoGen). The treatments were administered to mice on day 0 and 14. Blood samples were drawn from the tail vein on day 21.

Synthesis of peptides

All peptides were manufactured by GL Biochem (Shanghai) Ltd in the form of a dry powder. The peptides were generated using solid phase synthesis methods and the quality of the products was monitored by mass spectrometry. Three groups of peptides (106-160, 365-374, and 687-741) of the S protein could not be synthesised by this current method. All peptides were dissolved in pH 7.4 PBS buffer or 8 M pH

7.0 urea Na_2HPO_4/NaH_2PO_4 buffer. Peptides conjugated to keyhole limpet haemocyanin (KLH) carrier proteins were also purchased from GL Biochem (Shanghai) Ltd. These peptide-conjugated proteins were dissolved in a pH 7.4 PBS buffer.

Antibody detection using ELISA

Epitope-specific antibodies were detected by enzyme-linked immunosorbent assay (ELISA). Briefly, all peptides or recombinant proteins at a final concentration of 0.5 μg/mL in 50 mM coating buffer (pH 9.6 $Na_2CO_3/NaHCO_3$) were coated on ELISA plates (Nunc, Roskilde, Denmark) and incubated overnight at 4°C. Plates were blocked with TBS-5% (w/v) non-fat milk for 3 h at 37°C and washed four times in 0.05% Tween-20 (Sigma) in TBST. Diluted patient or mice sera were added into the wells and incubated for 1 h at 37°C. Plates were washed six times in TBS-0.05% Tween and incubated with horseradish peroxidase (HRP)-conjugated goat anti-mouse IgG (ThermoFisher, Catalog # 31410), goat anti-human IgM (ThermoFisher, Catalog # A18841), or goat anti-human IgA (ThermoFisher, Catalog # A18781) for 1 h at 37°C. The colour was developed using trimethyl borane (TMB) solution (Sigma) and absorbance was measured at 450 nm using an ELISA reader. Samples from non-immunised mice or healthy volunteers were used as controls. The cut-off lines were based on the mean value plus three times the standard deviation.

Identification of T cell epitopes using ELISpot

The T cell responses were detected by enzyme-linked immunosorbent spot (ELISpot) kits (Dakewe Biotech Co., Ltd) following the manufacturer's protocol. Briefly, splenocytes harvested from sacrificed mice were washed and immediately transferred to anti-IFN-γ antibody pre-coated filter plates. For stimulation, splenocytes were co-incubated with distinct epitopes overnight at 37℃. All samples were assayed with positive controls (Phorbol myristate acetate and Ionomycin) and cells from a reference donor. All images in different wells were captured using a CTL ImmunoSpot ELISpot Analyzer and processed using the ImmunoCapture software (Cellular Technology Ltd., USA).

Statistical analysis

ELISA data were collected on a Thermo Scientific VARIOSKAN FLASH 3001

(Ref:5250040). ELISpot assay results were captured using a CTL ImmunoSpot ELISpot Analyzer and processed using the ImmunoCapture software (Cellular Technology Ltd., USA). Data were reported as the median (indicating the range from minimum to maximum) and arithmetic mean ± standard deviation using, R software package version 3.4.1 and Microsoft office 365 Excel. All R-squared and t-test were calculated by Student's t-test using Microsoft office 365 Excel. All results were plotted in Prism 7 (GraphPad Software Inc., CA).

Expression and purification of recombinant protein

The genes encoding the spike RBD (amino acid residues 306 to 543 of the spike protein) and full-length NP of SARS-CoV-2 were codon-optimized using E. coli. Detailed information on the two recombinant proteins can be found in our previous work[1].

Microneutralisation tests

Serial two-fold dilutions of heat-inactivated sera (treated at 56ºC for 30 min) were prepared from a starting dilution of 1:10. The serum dilutions were mixed with equal volumes of 100 TCID50 (median tissue culture infective dose) of SARS-CoV-2 as indicated. After 1 h of incubation at 37ºC, 35 µL of the virus–serum mixture was added to a monolayer of Vero-E6 cells for SARS-CoV-2 infection in 96-well microtitre plates in quadruplicate. After 1 h of adsorption, an additional 150 µL of culture medium was added to each well and incubated for 3 days at 37ºC in 5% CO2 in a humidified incubator. A virus back-titration was performed without immune serum to assess the input virus dose. The cytopathic effect (CPE) was read at 3 days post infection. The highest serum dilution that completely protected cells from CPE in half the wells was estimated using the Reed-Muench method and was taken as the neutralising antibody titre. Positive and negative control sera were included to validate the assay.

Statistical analysis

ELISA data were collected on a Thermo Scientific VARIOSKAN FLASH 3001 (Ref:5250040). ELISpot assay results were captured using a CTL ImmunoSpot ELISpot Analyzer and processed using the ImmunoCapture software (Cellular Technology Ltd., USA). Data were reported as the median (indicating the range from

minimum to maximum) and arithmetic mean ± standard deviation using, R software package version 3.4.1 and Microsoft office 365 Excel. All R-squared and t-test were calculated by Student's t-test using Microsoft office 365 Excel. All results were plotted in Prism 7 (GraphPad Software Inc., CA).

Supplementary information, Fig. S1

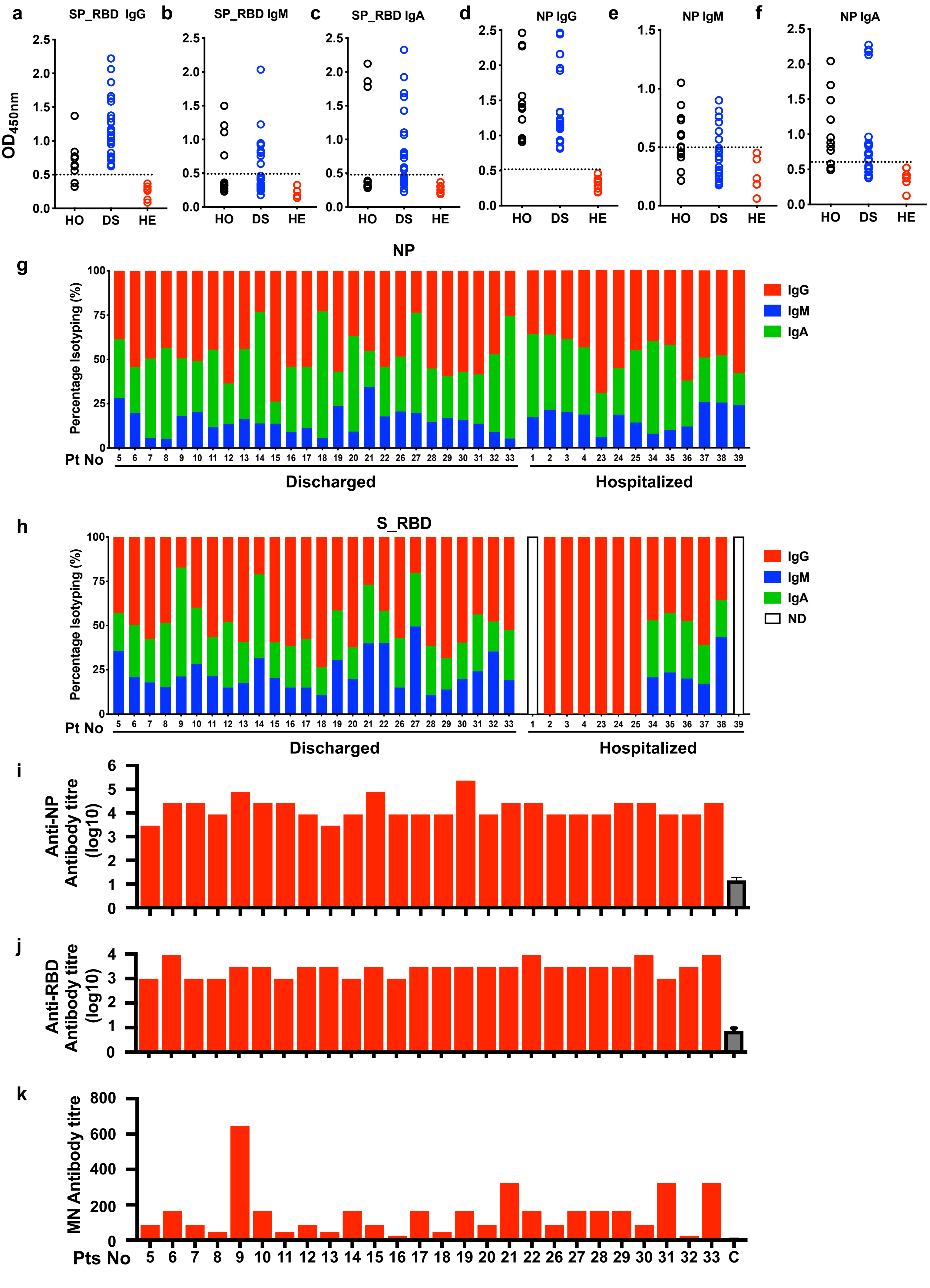

Supplementary information, Fig. S1 Detection of specific antibodies of SARS-CoV-2 proteins in early convalescent sera from COVID-19 patients by ELISA. a-c The recombinant SP_RBD protein was used as the coated antigen. **d-f** The recombinant N protein was used as the coated antigen. **g-h** Antibody isotyping of N and SP_RBD binding antibodies from COVID-19 patients. Sera from 26 discharged patients, 13 hospitalized patients, and 6 healthy blood donors were tested at a dilution of 1:100. The dashed lines represent cut-off values (the mean absorbance at 450 nm of sera from healthy blood donors plus three times the standard deviation). **i-k** N protein or RBD fragment of S protein-specific IgG levels and microneutralisation (MN) assay results of recovered patients' antibody titres. HO: Hospitalized patients' sera, DS: Discharged patients' sera, HE: Healthy donors' Sera, ND: Not detected or lower than cut-off values.

Supplementary information, Fig. S2

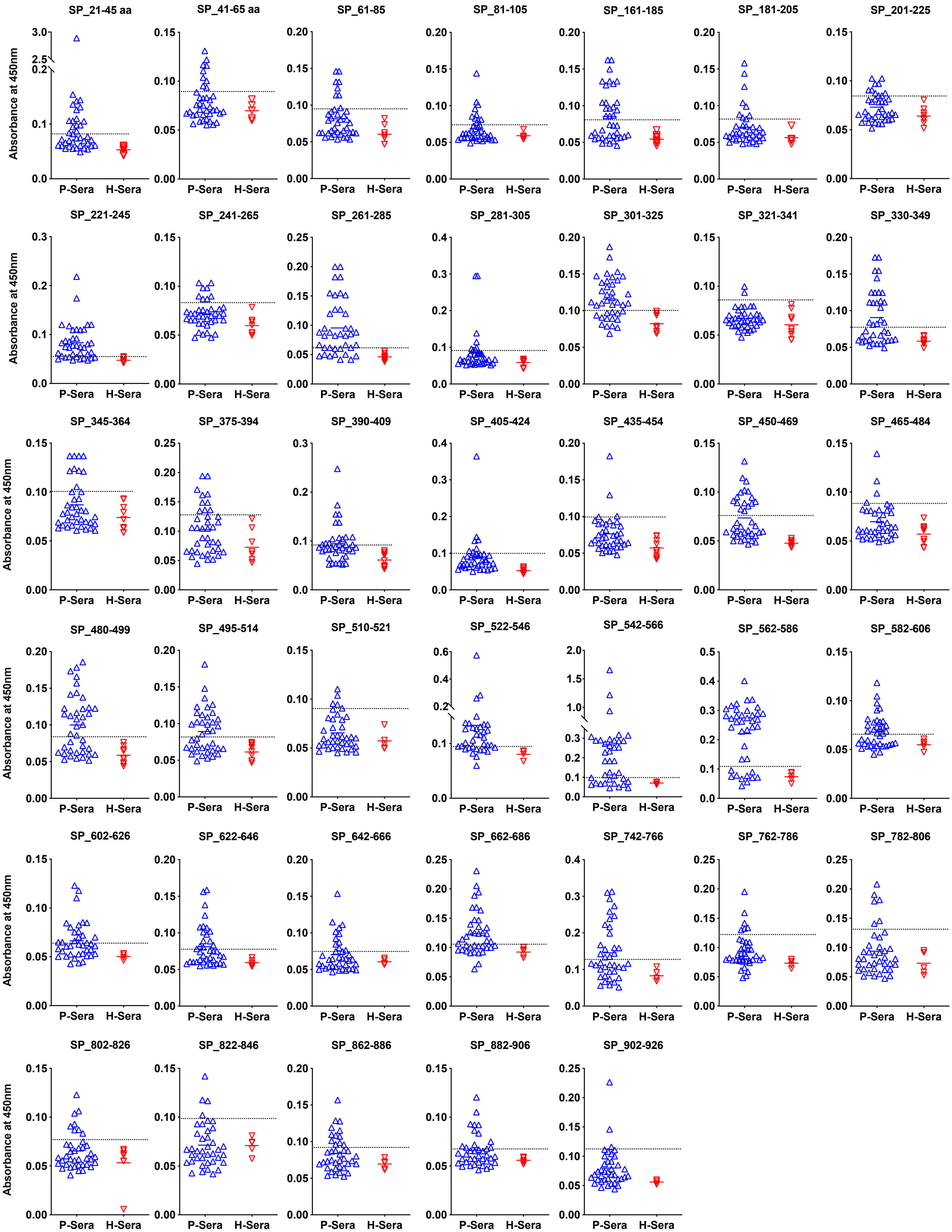

Supplementary information, Fig. S2 Detection of specific antibodies for S protein mapping of peptides by ELISA. Sera from 26 discharged patients, 13 hospitalized patients, and six healthy blood donors were tested at a dilution of 1:100. The dashed lines represent cut-off values (the mean absorbance at 450 nm plus three times the standard deviation in sera from healthy blood donors).

Supplementary information, Fig. S3

a

```
SARS-CoV-2  1     MFVFLVLLPLVSSQCVNLTTRTQL----PPAYT--NSFTRGVYYPDKVFRSSVLHSTQDL  54
                  MF+FL  L L S    +L   T        P YT    S  RGVYYPD +FRS  L  TQDL
SARS-CoV    1     MFIFLLFLTLTSGS--DLDRCTTFDDVQAPNYTQHTSSMRGVYYPDEIFRSDTLYLTQDL  58
...

SARS-CoV-2  175   FLMDLEGKQGNFKNLREFVFKNIDGYFKIYSKHTPINLVRDLPQGFSALEPLVDLPIGIN  234
                  F +D   K GNFK LREFVFKN DG+  +Y     PI+ VRDLP GF  L P    LP GIN
SARS-CoV    168   FSLDVSEKSGNFKHLREFVFKNKDGFLYVYKGYQPIDVVRDLPSGFNTLKPIFKLPLGIN  227

SARS-CoV-2  235   ITRFQTLLALHRSYLTPGDSSSGWTAGAAAYYVGYLQPRTFLLKYNENGTITDAVDCALD  294
                  IT F   L        P      W   AAAY+VGYL P TF+LKY+ENGTITDAVDC   +
SARS-CoV    228   ITNFRAILTA----FSPAQDI--WGTSAAAYFVGYLKPTTFMLKYDENGTITDAVDCSQN  281

SARS-CoV-2  295   PLSETKCTLKSFTVEKGIYQTSNFRVQPTESIVRFPNITNLCPFGEVFNATRFASVYAWN  354
                  PL E KC  KSF ++KGIYQTSNFRV P    +VRFPNITNLCPFGEVFNAT F SVYAW
SARS-CoV    282   PLAELKCSVKSFEIDKGIYQTSNFRVVPSGDVVRFPNITNLCPFGEVFNATKFPSVYAWE  341

SARS-CoV-2  355   RKRISNCVADYSVLYNSASFSTFKCYGVSPTKLNDLCFTNVYADSFVIRGDEVRQIAPGQ  414
                  RK ISNCVADYSVLYNS  FSTFKCYGVS TKLNDLCF NVYADSFV+ GD+VRQIAPGQ
SARS-CoV    342   RKKISNCVADYSVLYNSTFFSTFKCYGVSATKLNDLCFSNVYADSFVVKGDDVRQIAPGQ  401

SARS-CoV-2  415   TGKIADYNYKLPDDFTGCVIAWNSNNLDSKVGGNYNYLYRLFRKSNLKPFERDISTEIYQ  474
                  TG IADYNYKLPDDF GCV AWN   N D     GNYNY YR  R    L PFERDIS    +
SARS-CoV    402   TGVIADYNYKLPDDFMGCVLAWNTRNIDATSTGNYNYKYRYLRHGKLRPFERDISNVPFS  461

SARS-CoV-2  475   AGSTPCNGVEGFNCYFPLQSYGFQPTNGVGYQPYRVVVLSFELLHAPATVCGPKKSTNLV  534
                      PC       NCY PL   YGF   T G+GYQPYRVVVLSFELL APATVCGPK ST+L+
SARS-CoV    462   PDGKPCTP-PALNCYWPLNDYGFYTTTGIGYQPYRVVVLSFELLNAPATVCGPKLSTDLI  520

SARS-CoV-2  535   KNKCVNFNFNGLTGTGVLTESNKKFLPFQQFGRDIADTTDAVRDPQTLEILDITPCSFGG  594
                  KN CVNFNFNGLTGTGVLT S K F PFQQFGRD+ D TD VRDP T EILDI PC FGG
SARS-CoV    521   KNQCVNFNFNGLTGTGVLTPSSKRFQPFQQFGRDVSDFTDSVRDPKTSEILDISPCAFGG  580

SARS-CoV-2  595   VSVITPGTNTSNQVAVLYQDVNCTEVPVAIHADQLTPTWRVYSTGSNVFQTRAGCLIGAE  654
                  VSVITPGTN S +VAVLYQDVNCT+V  AIHADQLTP WR+YSTG NVFQT AGCLIGAE
SARS-CoV    581   VSVITPGTNASSEVAVLYQDVNCTDVSTAIHADQLTPAWRIYSTGNNVFQTQAGCLIGAE  640
...

SARS-CoV-2  895   QIPFAMQMAYRFNGIGVTQNVLYENQKLIANQFNSAIGKIQDSLSSTASALGKLQDVVNQ  954
                  QIPFAMQMAYRFNGIGVTQNVLYENQK IANQFN AI  IQ+SL  T  ALGKLQDVVNQ
SARS-CoV    877   QIPFAMQMAYRFNGIGVTQNVLYENQKQIANQFNKAISQIQESLTTTSTALGKLQDVVNQ  936
```

– CR3022 – B38 – S309 – m396 – 80R – F26G19

Glycosylation site; ↑ Prominent Alteration; SARS-CoV-2 ID; △ ACE2

b

c

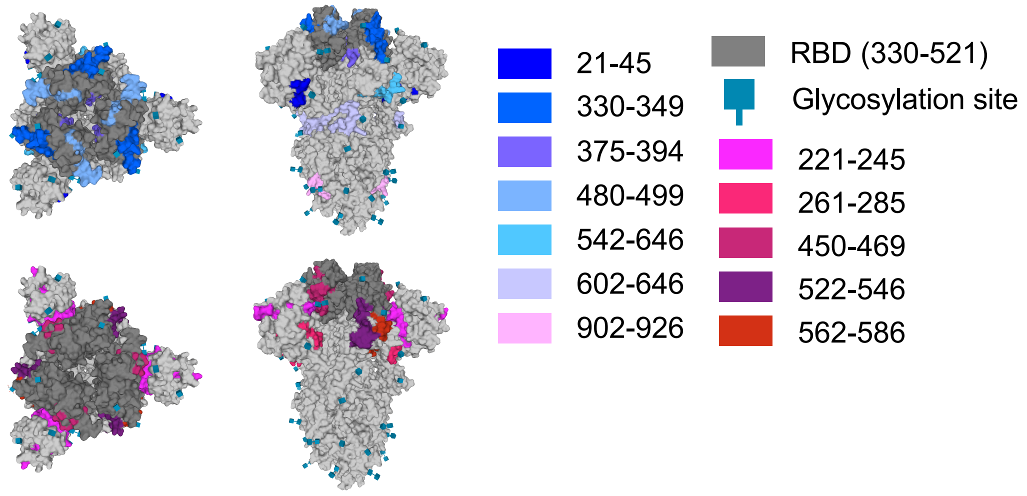

Supplementary information, Fig. S3 Structural representation of immunodominant and antibody binding sites in SARS-CoV-2 RBD. a Amino acid sequence alignment of SARS-CoV-2 and SARS-CoV RBD sequences. Related antibody binding sites, ACE2 binding sites, prominent alterations, and immunodominant sites of SARS-CoV-2 and SARS-CoV are shown. CR3022, a monoclonal antibody targeting a highly conserved cryptic epitope[2], had 14 out of 28 binding residues of the SARS-CoV-2 RBD located in ID sites; B38, a SARS-CoV-2 specific neutralising antibody, had 16 out of 34 residues in ID sites[3]; Another human antibody, S309, which can neutralise SARS-CoV-2 and SARS-CoV, had 11 out of 21 binding residues in ID sites[4]; F26G19, a mouse antibody, had 11 out of 20 residues in ID sites, likely indicating a relatively high binding affinity to the RBD fragment[5]; whereas m396, an antibody with relatively low binding affinity to SARS-CoV-2 RBD, only had 5 out of 22 binding residues located in ID sites[6]; and, R80, another low binding affinity antibody, had no matching binding residues[6] **b** Top view and side view of the S protein with immunodominant sites with positive rates ranging from 50% to 60%. **c** Top view and side view of the S protein with immunodominant sites with positive rates greater than 60%.

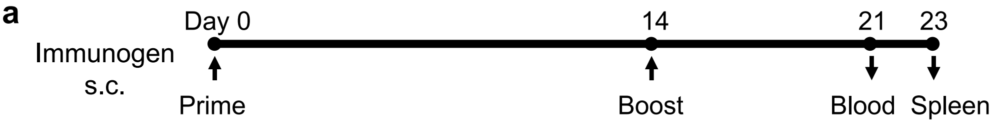

b

Group	Dose	Adjuvant
rRBD	25 µg	AHG
S370-395 - KLH	25 µg	AHG
S435-479 - KLH	25 µg	AHG
PBS buffer	-	AHG

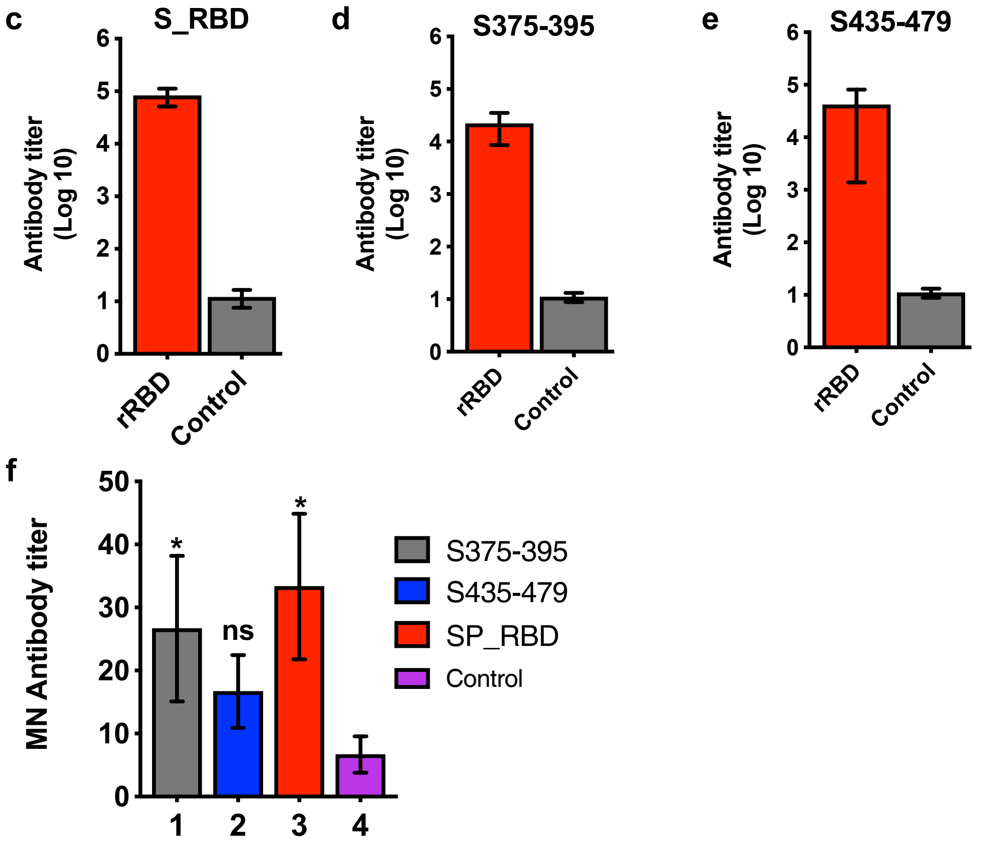

Supplementary information, Fig. S4 RBD-specific antibody responses in immunized mice. **a-b** Schematic representation of the mouse immunisation schedule. Balb/C mice (n = 5 per group) were immunised subcutaneously (s.c.) with 25 µg of rRBD and KLH-conjugated peptides S370-395 and S435-479 mixed with aluminium hydroxide gel (AHG). **c-e** rRBD-specific antibody responses in immunised mice. rRBD-specific IgG antibody responses in mouse sera collected at 7 days after the second vaccination. **f** Virus microneutralisation (MN) titres were measured against SARS-CoV-2 in classical BSL3. The ELISA assay showed that mice immunised with the entire RBD, S370-395, and S435-479 generated high levels of specific antibodies with titres over 10^4, while the MN test showed mice immunised with S370-395, S435-479, or RBD had viral neutralising titres of 1:26.7, 1:16.7, and 1:33.3, respectively.

Supplementary information, Table S1 List of synthesized peptides.

Position	Sequence
21-45	RTQLPPAYTNSFTRGVYYPDKVFRS
41-65	KVFRSSVLHSTQDLFLPFFSNVTWF
61-85	NVTWFHAIHVSGTNGTKRFDNPVLP
81-105	NPVLPFNDGVYFASTEKSNIIRGWI
161-185	SSANNCTFEYVSQPFLMDLEGKQGN
181-205	GKQGNFKNLREFVFKNIDGYFKIYS
201-225	FKIYSKHTPINLVRDLPQGFSALEP
221-245	SALEPLVDLPIGINITRFQTLLALH
241-265	LLALHRSYLTPGDSSSGWTAGAAAY
261-285	GAAAYYVGYLQPRTFLLKYNENGTI
281-305	ENGTITDAVDCALDPLSETKCTLKS
301-325	CTLKSFTVEKGIYQTSNFRVQPTES
321-341	QPTESIVRFPNITNLCPFGEV
330-349	PNITNLCPFGEVFNATRFAS
345-364	TRFASVYAWNRKRISNCVAD
375-394	STFKCYGVSPTKLNDLCFTN
390-409	LCFTNVYADSFVIRGDEVRQ
405-424	DEVRQIAPGQTGKIADYNYK
420-439	DYNYKLPDDFTGCVIAWNSN
435-454	AWNSNNLDSKVGGNYNYLYR
450-469	NYLYRLFRKSNLKPFERDIS
465-484	ERDISTEIYQAGSTPCNGVE
480-499	CNGVEGFNCYFPLQSYGFQP
495-514	YGFQPTNGVGYQPYRVVVLS
510-521	VVVLSFELLHAP
522-546	ATVCGPKKSTNLVKNKCVNFNFNGL
542-566	NFNGLTGTGVLTESNKKFLPFQQFG
562-586	FQQFGRDIADTTDAVRDPQTLEILD
582-606	LEILDITPCSFGGVSVITPGTNTSN
602-626	TNTSNQVAVLYQDVNCTEVPVAIHA
622-646	VAIHADQLTPTWRVYSTGSNVFQTR
642-666	VFQTRAGCLIGAEHVNNSYECDIPI
662-686	CDIPIGAGICASYQTQTNSPRRARS
742-766	ICGDSTECSNLLLQYGSFCTQLNRA
762-786	QLNRALTGIAVEQDKNTQEVFAQVK
782-806	FAQVKQIYKTPPIKDFGGFNFSQIL
802-826	FSQILPDPSKPSKRSFIEDLLFNKV
822-846	LFNKVTLADAGFIKQYGDCLGDIAA
842-866	GDIAARDLICAQKFNGLTVLPPLLT
862-886	PPLLTDEMIAQYTSALLAGTITSGW
882-906	ITSGWTFGAGAALQIPFAMQMAYRF
902-926	MAYRFNGIGVTQNVLYENQKLIANQ

References

1. To, K.K.-W., *et al.* Temporal profiles of viral load in posterior oropharyngeal saliva samples and serum antibody responses during infection by SARS-CoV-2: an observational cohort study. *The Lancet Infectious Diseases* (2020).
2. Yuan, M., *et al.* A highly conserved cryptic epitope in the receptor-binding domains of SARS-CoV-2 and SARS-CoV. *Science* (2020).
3. Wu, Y., *et al.* A noncompeting pair of human neutralizing antibodies block COVID-19 virus binding to its receptor ACE2. *Science*, eabc2241 (2020).
4. Pinto, D., *et al.* Cross-neutralization of SARS-CoV-2 by a human monoclonal SARS-CoV antibody. *Nature* (2020).
5. Park, T., *et al.* Spike protein binding prediction with neutralizing antibodies of SARS-CoV-2. *bioRxiv* (2020).
6. Tian, X., *et al.* Potent binding of 2019 novel coronavirus spike protein by a SARS coronavirus-specific human monoclonal antibody. *Emerging microbes & infections* **9**, 382-385 (2020).
